# ‘Setting the Benchmark’ Part 1: The Contextualised Physical Demands of Positional Roles in the FIFA World Cup Qatar 2022

**DOI:** 10.5114/biolsport.2024.131090

**Published:** 2023-09-07

**Authors:** Paul S. Bradley

**Affiliations:** 1 FIFA, Zürich, Switzerland

**Keywords:** Match analysis, High-intensity, Playing position, International football, Soccer

## Abstract

This study aimed to contextualise and benchmark the positional role demands during the FIFA World Cup Qatar 2022. With FIFA’s official approval, all sixty-four games were analysed during the competition (n=722) using a multi-camera computerised tracking system. During a typical FIFA World Cup Qatar 2022 match, defensive and central midfielders covered 8-19% more total distance than other positional roles (P<0.01; Effect Size [ES]: 0.8-2.5). The distances covered at higher intensities (≥20 and ≥25 km · h^-1^) were 16–92% and 36–138% higher for wide midfielders and wide forwards compared to central defenders, defensive and central midfielders as well as centre forwards (*P*<0.01; ES: 0.7–2.2 and ES: 0.6–1.4). Defensive and central midfielders covered a greater proportion of their distance at higher intensities (≥20 and ≥25 km · h^-1^) out-of-possession (71–83%; *P*<0.01; ES: 1.4–3.0), whilst attacking midfielders, wide and centre forwards more in-possession (55–68%; *P*<0.01; ES: 1.6–3.3). Nine out of the top ten sprint speeds attained at the tournament were from wide positional roles (35.3–35.7 km · h^-1^). All positional roles demonstrated a second half reduction in total distance covered compared to the first half (*P*<0.01; ES: 0.8–1.3). A decline between halves for the distances covered at higher intensities (≥20 and ≥25 km · h^-1^) were more evident in attacking midfielders, wide defenders and midfielders than for other positional roles (*P*<0.01; ES: 0.3–0.7). Defensive midfielders and centre forwards were found to have the highest coefficient of variation (CV: 30.9–35.9% and 67.7–67.8%) for the distances covered at higher intensities (≥20 and ≥25 km · h^-1^) compared to other positional roles. The current findings provide valuable contextual information about the contemporary positional demands of international football. This could be useful in the development and prescription of specific training regimes for national teams.

## INTRODUCTION

Match analysis research can be effectively used to benchmark the demands of match-play, whilst also providing a conceptual framework for the development of specific performance tests and training regimes [1]. Authors have documented the match demands of the leading domestic leagues around the world [2–4], in addition to some European competitions [5]. However, there is limited information on the match demands from contemporary international football tournaments such as the FIFA World Cup. Studies conducted to date have typically used freely available data that had limited metric and positional granularity and were performed on the outdated FIFA World Cup Brazil 2014 [6]. Therefore, a study that was officially approved by FIFA and thus included all metrics and player observations from the FIFA World Cup Qatar 2022 could provide a more accurate depiction of the current demands of international match-play. Since there is extensive variability in the match demands both between and within players according to match and positional role [7–8], it is imperative to benchmark such recent competitions.

A consistent finding in the football science literature is that the match demands of players are highly dependent upon their unique positional role in the team [3, 9]. Most studies use broad categories to define outfield roles such as defenders, midfielders and forwards or even distinct sub-categories within them (e.g., midfielders can be split into central and wide midfielders). Recent findings demonstrated that the match demands of specialised outfield roles in the English Premier League resulted in highly distinguishable movement characteristics compared to more broad categories [10]. Although no research to date has partitioned international players into highly specialised outfield positions in tournaments such as the FIFA World Cup Qatar 2022. This would be highly relevant to the football community especially given the up-to-date nature of this data and its comprehensive positional breakdown. To enable trends across specialised positional roles to be translated more effectively to scientists and practitioners, the author has attempted to layer context throughout the analysis to add a narrative to the numbers [11]. This contextualisation is unlike any published article in this field, as it will highlight with the permission of FIFA distinct individualised examples of positional profiles at the upper and lower extremes to add more context to the trends.

Some researchers have suggested that distances covered at the higher intensities in matches are valid measures of physical performance in football [12], and are a distinguishing characteristic between different standards of players [13–14]. Thus, high-intensity metrics seem important to consider as they have evolved significantly over the last decade [2, 3, 15], and are involved in game-changing moments such as goalscoring opportunities [16]. Research has consistently found that high-intensity metrics were reduced in the second versus the first half of games [1]. However, limited information has been published on half-by-half deficits in recent international tournaments. This is particularly relevant as recent rule changes regarding added time have resulted in much longer game durations in the FIFA World Cup Qatar 2022. Thus, a detailed examination of high-intensity metrics across halves would provide valuable information regarding the distribution of positional work rates in a modified game format (e.g., FIFA accounting for all stoppages). Therefore, this study aimed to contextualise and benchmark the positional role demands during the FIFA World Cup Qatar 2022.

## MATERIALS AND METHODS

### Sample

With FIFA’s official approval, all 64 games during the FIFA World Cup Qatar 2022 were collected and analysed. Analyses involved the examination of the match physical performances of individual players in various positional roles in the team. The data provider assigned eight different outfield roles to enable positional differences to be determined. Games were filtered so only players who completed the entire match were evaluated. Moreover, filters ensured that all match data were collected over the duration of a normal match or regular time plus added time, but no extra-time data were included. This allowed profiling of 722 player observations in various positional roles (219 central defenders [CD], 174 wide defenders [WD], 68 defensive midfielders [DM], 100 central midfielders [CM], 21 attacking midfielders [AM], 49 wide midfielders [WM], 48 wide forwards [WF], and 43 centre forwards [CF]). As this data are freely available [17], no ethical approval was required.

### Match Analysis System

All FIFA World Cup Qatar 2022 games were analysed using a multicamera computerised tracking system (TRACAB, Chyron Hego). All player movements were captured by high-definition cameras operating at 25 Hz. This systems validity has been quantified to verify the capture process and subsequent accuracy of the data [18]. After system calibration and various stringent quality control processes, the data captured were analysed using match analysis software. This produced a data set on each player’s activity pattern during a match using specified speed zones.

### Speed Zones

Players’ activities were coded into the following:

- Zone 1 (0.0–6.9 km · h^-1^),
- Zone 2 (≥7.0–14.9 km · h^-1^),
- Zone 3 (≥15.0–19.9 km · h^-1^),
- Zone 4 (≥20–24.9 km · h^-1^),
- Zone 5 (≥25.0 km · h^-1^).

Total distance represented the sum of the distances covered above. High-intensity activity consisted of the aggregation of Zones 4 and 5 (≥20.0 km · h^-1^), whilst sprinting exclusively included Zone 5 activity (≥25.0 km · h^-1^). Similar classifications have been employed in elite football for over a decade [13]. Moreover, these speed demarcations were also employed at the 2018 FIFA World Cup Russia [19]. Maximum sprint speeds were also quantified across all positional roles.

### Statistical Analyses

All statistical analyses were conducted using SPSS (SPSS Inc., Chicago, USA). Descriptive statistics were calculated on each variable and z-scores were used to verify normality. Differences across positional roles and halves were determined using factorial analysis of variance (ANOVA). In the event of a significant difference, the appropriate post-hoc tests were used to identify any localised effects. Statistical significance was set at *P* < 0.05. The coefficient of variation (CV) was calculated to determine the data spread across each metric. Effect sizes (ES) were computed to determine the meaningfulness of any differences and corrected for bias using Hedges formula. The ES magnitudes were classed as trivial (< 0.2), small (> 0.2–0.6), moderate (> 0.6–1.2) and large (> 1.2). Pearson’s coefficients were used for correlation analyses and the magnitudes of the associations were regarded as trivial (*r* ≤ 0.1), small (*r* > 0.1–0.3), moderate (*r* > 0.3–0.5), large (*r* > 0.5–0.7), very large (*r* > 0.7–0.9), and nearly perfect (*r* > 0.9). Values are presented as means and standard deviations unless otherwise stated.

## RESULTS

### Benchmarking & Variation

The data presented in Figures 1A-D benchmarked each positional role from a physical perspective. Figure 1A highlights that CM and DM covered 8–19% more total distance than other positional roles (*P* < 0.01; ES: 0.8–2.5). Figure 1A also demonstrates that attacking positional roles such as AM, WF and CF have the highest coefficient of variation in the total distance covered (CV: 10.2–12.0%) compared to more defensive positional roles such as CD, WD, DM and CM (CV: 6.7–6.9%). Figures 1B and 1C illustrate the distances covered at higher intensities (≥20 and ≥25 km · h^-1^, respectively) were 16–92% and 36–138% higher in wide positional roles such as WM and WF compared to central positional roles such as CD, DM, CM and CF (*P* < 0.01; ES: 0.7–2.2 and 0.6–1.4). Figures 1B and 1C illustrates that DM and CF have the highest coefficient of variation (CV: 30.9–35.9% and 67.7–67.8%) for the distances covered at higher intensities (≥20 and ≥25 km · h^-1^, respectively) compared to WM, AM and WF (CV: 23.1–26.2% and 43.2–50.7%). Top sprint speeds were faster during games for WD, AM, WM, WF and CF (32.1–32.3 km · h^-1^), compared to CD, DM and CM (30.2–30.8 km · h^-1^; *P* < 0.01; ES: 0.8–1.1). Figure 1D demonstrates that the top ten sprint speeds attained at the tournament were primarily from wide positions with WF (40%), WD (30%) and WM (20%), accounting for 90% of these efforts. The coefficient of variation of these top ten sprint efforts varied across wide roles (WF = 3.5–6.0%, WD = 2.8–5.8%, WM = 1.0–2.4%).

**FIG. 1A:**
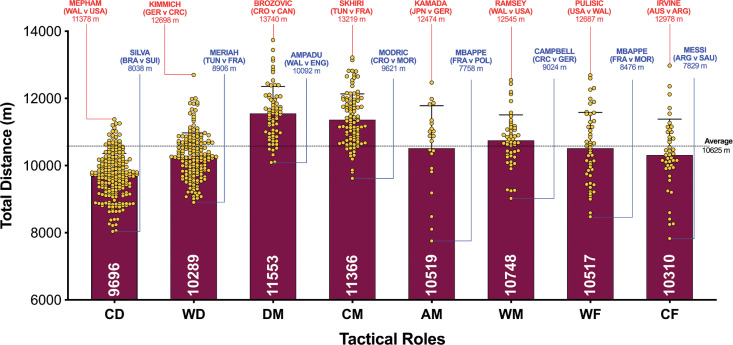
Total Distance and variation for each positional role in the Qatar FIFA World Cup 2022. Data normalized for 90+ min (excludes GK and extra time). CD = central defender, WD = wide defender, DM = defensive midfielder, CM = central midfielder, AM = attacking midfielder, WM = wide midfielder, WF = wide forward, CF = centre forward. Red = max, Blue = min.

**FIG. 1B:**
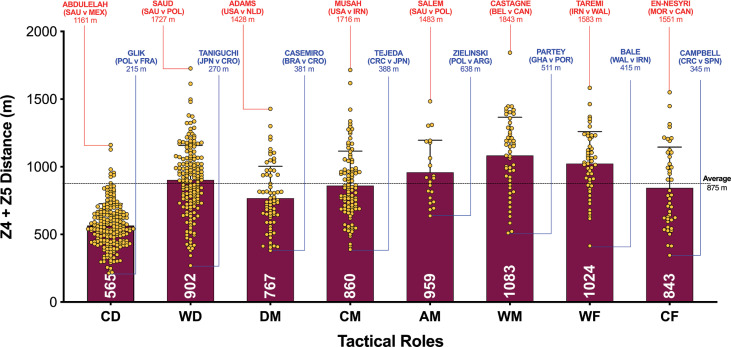
High Intensity Distance (≥20 km · h^-1^; Zone 4 and 5; Z4+Z5) and variation for each positional role in the Qatar FIFA World Cup 2022. Data normalized for 90+ min (excludes GK and extra time). CD = central defender, WD = wide defender, DM = defensive midfielder, CM = central midfielder, AM = attacking midfielder, WM = wide midfielder, WF = wide forward, CF = centre forward. Red = max, Blue = min.

**FIG. 1C:**
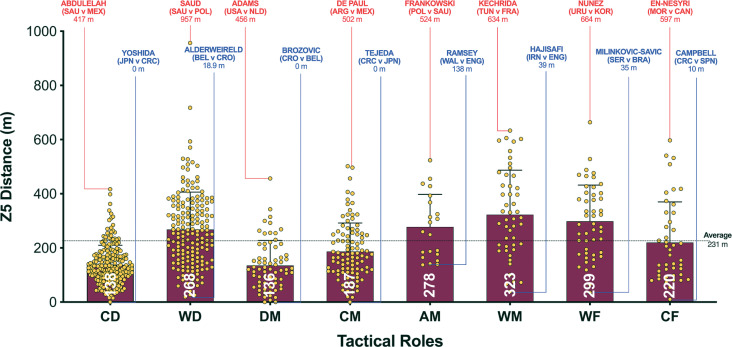
Sprint Distance (≥25 km · h^-1^; Zone 5; Z5) and positional variation in the Qatar FIFA World Cup 2022. Data normalized for 90+ min (excludes GK and extra time). CD = central defender, WD = wide defender, DM = defensive midfielder, CM = central midfielder, AM = attacking midfielder, WM = wide midfielder, WF = wide forward, CF = centre forward. Red = max, Blue = min.

**FIG. 1D:**
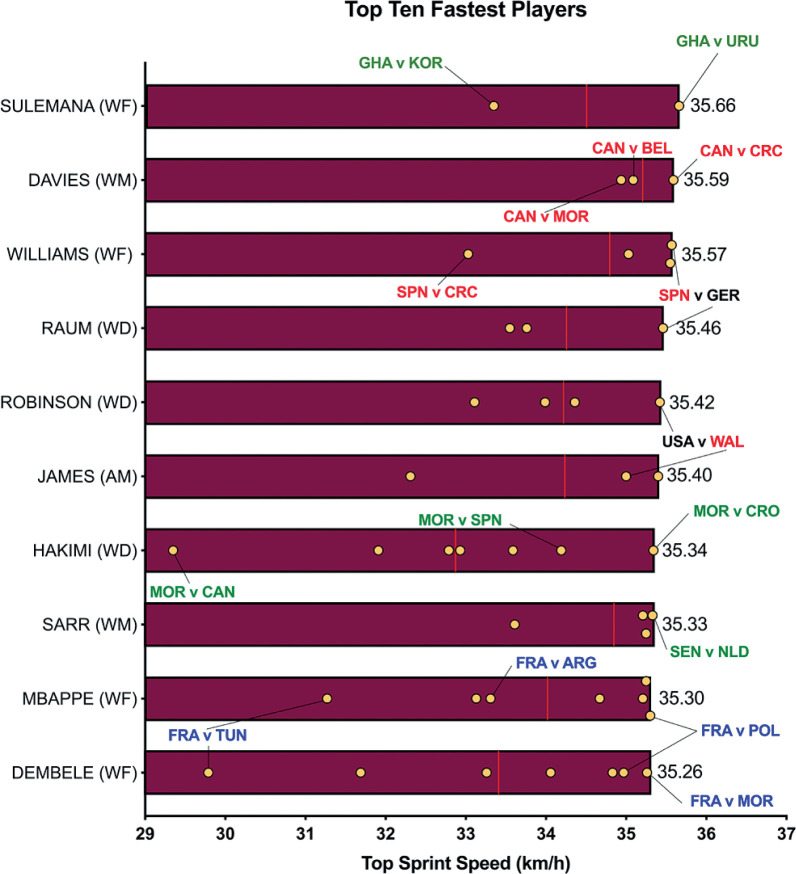
Ten Top Speeds and match-to-match variation in the Qatar FIFA World Cup 2022 (yellow dots signify each game and red line is the average). Data are not normalized for 90+ min and each players positional role is indicated in parentheses. WD = wide defender, AM = attacking midfielder, WM = wide midfielder, WF = wide forward. Text colour aligns with each position’s national colours.

### Quadrant Plots

The data presented in Figures 2A-C correlates two distinct dimensions of physical performance using quadrant plots to compare the specialist defensive, midfield and attacking positional roles against one another. Figure 2A demonstrates a moderate association between a CD total and high-intensity game distances (*r* = 0.46; *P* < 0.01) and this positional role primarily occupied the lower-left quadrant (lower-left [LLQ] = 86%, lower-right [LRQ] = 10%, upper-left [ULQ] = 3% and upper-right quadrant [URQ] = 1%). A large association was observed between a WD total and high-intensity game distances (*r* = 0.51; *P* < 0.01), with most residing in the lower- or upper-left quadrants (LLQ = 40%, LRQ = 9%, ULQ = 30% and URQ = 21%). Large correlation coefficients were also found between a DM total and high-intensity game distances (*r* = 0.51; *P* < 0.01). DM were primarily split between the upper- and lower-right quadrants (LLQ = 9%, LRQ = 60%, ULQ = 0% and URQ = 31%).

**FIG. 2A:**
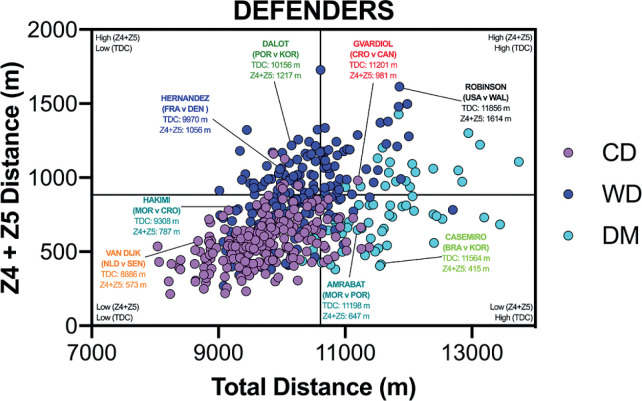
Defenders Total versus High Intensity Distance (≥20 km · h^-1^; Zone 4 and 5; Z4+Z5) in the Qatar FIFA World Cup 2022. Data normalized for 90+ min (excludes GK and extra time). Crosshairs were based on the average for all tactical roles. CD = central defender, WD = wide defender, DM = defensive midfielder. Text colour aligns with each position’s national colours.

**FIG. 2B:**
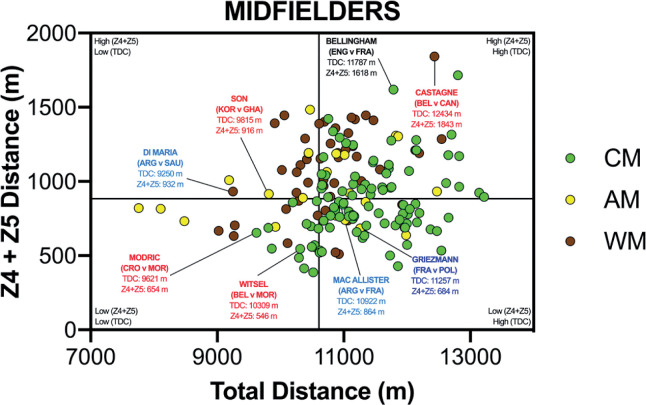
Midfielders Total versus High Intensity Distance (≥20 km · h^-1^; Zone 4 and 5; Z4+Z5) in the Qatar FIFA World Cup 2022. Data normalized for 90+ min (excludes GK and extra time). Crosshairs were based on the average for all tactical roles. CM = central midfielder, AM = attacking midfielder, WM = wide midfielder. Text colour aligns with each position’s national colours.

**FIG. 2C:**
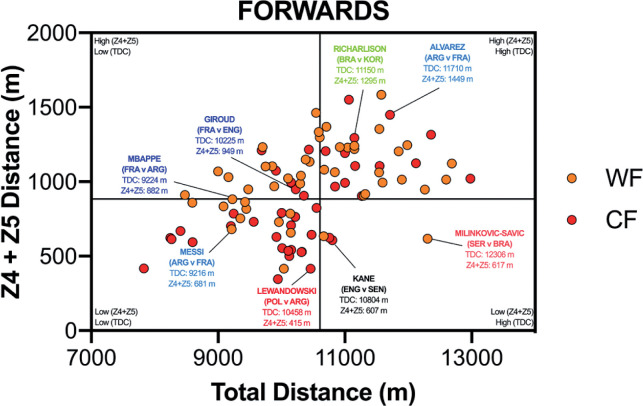
Forwards Total versus High Intensity Distance (≥20 km · h^-1^; Zone 4 and 5; Z4+Z5) in the Qatar FIFA World Cup 2022. Data normalized for 90+ min (excludes GK and extra time). Crosshairs were based on the average for all tactical roles. WF = wide forward, CF = centre forward. Text colour aligns with each position’s national colours.

Figure 2B visualises the specialist midfield positional roles, with CM and WM both demonstrating moderate associations between the total and high-intensity game distances (*r* = 0.31 and 0.45; *P* < 0.01). Whilst CM mainly occupied the upper- and lower-right quadrants (LLQ = 17%, LRQ = 43%, ULQ = 0% and URQ = 40%), WM were primarily located in the upper-right quadrant (LLQ = 15%, LRQ = 10%, ULQ = 20% and URQ = 55%). However, a small association was observed between an AM total and high-intensity game distances (*r* = 0.23; *P* > 0.05), and it was clear that they did not occupy any specific quadrant (LLQ = 19%, LRQ = 24%, ULQ = 24% and URQ = 33%).

Figure 2C highlights the most specialised offensive roles, with WF demonstrating a moderate association (*r* = 0.32; *P* < 0.05), whilst CF illustrated a large strength correlation between the total and high-intensity game distances (*r* = 0.58; *P* < 0.01). The WF were pre-dominantly spread across the upper-left and right quadrants (LLQ = 23%, LRQ = 4%, ULQ = 31% and URQ = 42%), whilst CF were principally located in the lower-left quadrant (LLQ = 53%, LRQ = 5%, ULQ = 14% and URQ = 28%). Both offensive positional roles rarely entered the lower-right quadrant.

### In-Possession & Out-of-Possession

Those players with a vital defensive duty in the team such as CD, WD, DM, CM and WM covered a greater proportion of their overall distance out-of-possession compared to more offensive positions such as WF (48–49% vs 44%; *P* < 0.01; ES: 0.6–0.8). In contrast, WF covered a higher proportion of their overall distance covered in-possession compared to CD, WD, AM and WM (48% vs 43%; *P* < 0.01; ES: 0.7–0.8). Defensive positional roles such as CD and DM cover a greater proportion of their distance at higher intensities (≥20 and ≥25 km · h^-1^) out-of-possession than offensive positional roles such as AM, WF and CF (71–77% vs 40–44%; *P* < 0.01; ES: 1.7–3.0 and 73–83% vs 31–37%; *P* < 0.01; ES: 1.4–3.0). In contrast, AM, WF and CF covered a higher proportion of their distance at higher intensities (≥20 and ≥25 km · h^-1^) in-possession compared to CD and DM (55–58% vs 21–28%; *P* < 0.01; ES: 1.8–3.3 and 62–68% vs 15–23%; *P* < 0.01; ES: 1.6–3.1).

### Half-by-Half Differences

Figures 3A-C highlight the half-by-half differences for each of the positional roles on a per min basis. Figure 3A illustrates that all positions demonstrated a second half reduction in total distance covered compared to the first half (*P* < 0.01; ES: 0.8–1.3). Figure 3B demonstrates that a decline between halves for high-intensity distance (≥20 km · h^-1^) was evident for WD, DM, CM, AM, WM and CF (*P* < 0.01; ES: 0.3–0.7) but not for CD and WF (*P* > 0.05; ES: 0.1–0.2). Figure 3C indicates that the decline between halves for sprinting distance (≥25 km · h^-1^) was evident for WD, AM and WM (*P* < 0.05; ES: 0.3–0.4) but not for CD, DM, CM, WF and CF (*P* > 0.05; ES: 0.0–0.2).

**FIG. 3A:**
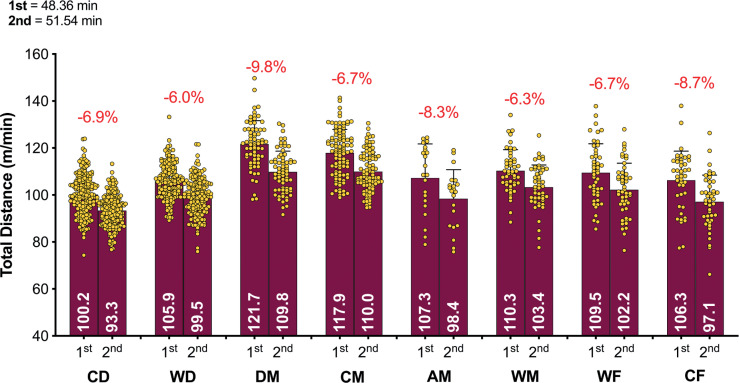
Positional half by half Total Distance in the Qatar FIFA World Cup 2022. Data normalized per min and only players completing 90+ min (excludes extra time). CD = central defender, WD = wide defender, DM = defensive midfielder, CM = central midfielder, AM = attacking midfielder, WM = wide midfielder, WF = wide forward, CF = centre forward. Red = a second half decline, Blue = no second half decline.

**FIG. 3B:**
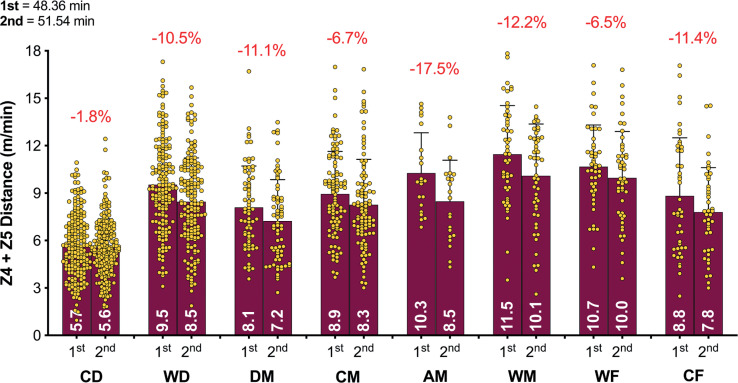
Positional half by half High Intensity Distance (≥20 km · h^-1^; Zone 4 and 5; Z4+Z5) in the Qatar FIFA World Cup 2022. Data normalized per min and only players completing 90+ min (excludes extra time). CD = central defender, WD = wide defender, DM = defensive midfielder, CM = central midfielder, AM = attacking midfielder, WM = wide midfielder, WF = wide forward, CF = centre forward. Red = a second half decline, Blue or Black = no second half decline.

**FIG. 3C:**
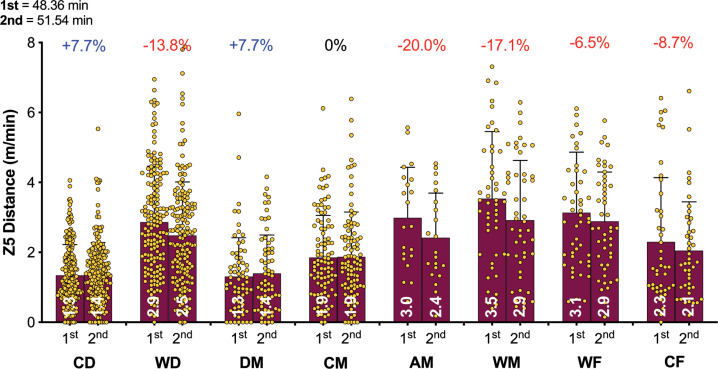
Positional half by half Sprint Distance (≥25 km · h^-1^; Zone 5; Z5) in the Qatar FIFA World Cup 2022. Data normalized per min and only players completing 90+ min (excludes extra time). CD = central defender, WD = wide defender, DM = defensive midfielder, CM = central midfielder, AM = attacking midfielder, WM = wide midfielder, WF = wide forward, CF = centre forward. Red = a second half decline, Blue or Black = no second half decline.

## DISCUSSION

This study was the first to contextualise and physically benchmark eight specialised positional roles during the FIFA World Cup Qatar 2022. Thus, this section will attempt to integrate the present findings with a contextual narrative to aid interpretation. Data revealed that central and defensive midfielders covered the greatest total distance and central defenders covered the lowest in the FIFA World Cup Qatar 2022. As similar findings have been documented in elite domestic football [13, 20], this current finding verifies that this trend also occurs at the highest standard of international football. Midfielders are renowned for their all-round industrious and versatile nature [2], and as such they covered the most ground during World Cup games. To further contextualise this trend, Croatia’s Brozović and Tunisia’s Skhiri covered > 13 km during World Cup games and thus set the upper benchmark for contemporary international midfielders. Midfielders have been found to be highly active during all phases of play [10]. Thus, unsurprisingly the present study found they covered the greatest in- and out-of-possession distances of the tournament. In contrast, central defenders are primarily active during out-of-possession phases [21], hence the lower total distance covered. However, due to the ever-changing demands placed on central defenders, they increasingly receive the ball from their goalkeeper to play out from the back or from teammates to (re)start build-up play [2, 10, 22]. To place this finding into a football context, it is imperative the reader is aware that the opposition’s quality and work rate can have a direct bearing on a central defenders’ activity profile [23]. For instance, Wales central defender Mepham covered > 11 km during the USA match, which was the upper benchmark for this positional role in the whole tournament. This can be attributed to the USA’s extreme work rate alongside their frequent progressions and final-third entries during this game (e.g., highest ranked team for overall and high-intensity distance covered, see Part 2 for more detail). In contrast, Brazilian central defender Silva covered the lowest total distance of 8 km for this positional role against Switzerland. Despite similar work-rate profiles between teams, this may have been due to Brazil’s attacking dominance throughout the match and the lack of threat from Switzerland, resulting in this sedate work-rate profile for Silva.

A focal point of researchers is usually the distances covered at higher intensities (≥20 km · h^-1^), due to its instrumental role in game-changing moments [16]. In the present study, wide positional roles, such as wide midfielders and forwards, clearly covered more distance at the higher intensities compared to players in central positional roles, such as central defenders and midfielders. This falls in line with a plethora of studies using elite domestic populations [2, 24], but scant evidence exists on international samples. In the present study, both wide positional roles attained an average high-intensity distance (≥20 km · h^-1^) of around 1.0–1.1 km. However, at the upper-level Belgium’s Castagne and Iran’s Taremi covered around 1.6–1.8 km, highlighting how demanding these wide roles are in the modern game. Some could attribute this finding to superior physical characteristics for wide versus central players [25], but it could simply be related to the extra space afforded to wide players along the flanks that enabled them to accelerate up to higher speeds when tactically required [11]. Wide players may also have been more active as teams utilised the flanks heavily in Qatar 2022 vs Russia 2018, as evidenced by a much greater number of goals from crosses (45 vs 25 goals). In contrast, central defenders and midfielders operate in pitch regions that are highly dense with players, which may limit their ability to accelerate into space at higher speeds when tactically required [2, 10]. This may have resulted in central positional roles covering much lower high-intensity distances during games of between 0.5–0.8 km. Data from the present study supports this assertation as the top ten fastest sprints in the FIFA World Cup Qatar 2022 were primarily by players in wide positional roles and they attained speeds > 35 km · h^-1^.

Translating match demands into positional drills is of the utmost importance to practitioners [26]. Precisely an overload stimulus that taxes the relevant physical attributes to enable players to fulfil their positional duties across the 90 min (volume) and during intensified periods (intensity). Thus, the current data were viewed from a unique perspective by correlating these two distinct dimensions of physical performance using quadrant plots. This may allow practitioners to determine which positional roles were more volume-based and which were more intensity-based by plotting the total distance against the distances covered at higher intensities (≥20 km · h^-1^). Unsurprisingly, central defenders and centre forwards primarily occupied the lower-left quadrant (53–85%), thus they generally exhibited low volume and intensity characteristics during games. Centre forwards and central defenders covered > 70% of their high-intensity distance in- and out-of-possession, respectively. Accordingly, the work rate profiles of these two positional adversaries are inextricably connected [27]. Interdependency is exhibited as centre forwards run into offensive areas to attack space or to run with the ball, whilst central defenders react through various runs to defensively press, cover or track back [9, 10–11]. Although one could extrapolate from such trends, the degree of physical preparation needed for these two roles, caution is still needed given the large coefficient of variation that exist in their physical profiles. This is especially evident for centre forwards, as 28% of the sample also resided in the upper-right quadrant (high volume and intensity), illustrating the greatest coefficient of variation across any positional role. Given such data spread, conditioning practices should always align with the age and capabilities of the players in these roles, in addition to the playing style(s) adopted by each nation [28]. To exemplify this point from a forward’s perspective, three of the greatest modern-day forwards fall within the lower-left quadrant (Messi, Mbappé & Lewandowski), in which the game model provides more freedom for them to be selective when exerting themselves. At the opposite end of the physical continuum, wide midfielders mainly occupied the upper-right quadrant (55%), exhibiting both volume and intensity characteristics. Research demonstrates a potential reason for this upper-right quadrant dominance, as they are highly active throughout games during both in-possession and out-of-possession phases (volume) and are also instrumental during defensive and offensive transitions (intensity) during peak game periods [9, 29]. Interestingly, central midfielders exhibit dual tendences as they occupied both the lower- and upper-right quadrants (40–43%), clearly highlighting their volume characteristics to support teammates in-possession but to also to press, cover and track back when out-of-possession [9, 11]. During intensified periods, some central midfielders exhibit box-to-box qualities and produce significant amounts of intensity to run with the ball, move to receive, support, attack the space in behind and to break into the box [29]. This is unlike more volume based defensive midfielders that were mainly found in the lower-right quadrant (60%), and were very active but mainly at low to moderate speeds (e.g., Zones 1–3). Whilst attacking midfielders did not occupy any specific quadrant and were uniformly spread across quadrants. Thus, it might be prudent for practitioners to adopt high-intensity training drills that not only mimic each positional role but also the individual characteristics of each player. This type of training has been found to tax both the aerobic and anaerobic systems and thus create desirable adaptation to develop players physiological capacities [30–32].

The distance covered in total and at higher intensities (≥20 km · h^-1^) generally declines from the first to the second half of a match [1, 14], although some studies have observed minimal half-by-half differences [13]. This trend is based on a reasonably similar duration played in the two halves. However, FIFA’s new approach to added time in the World Cup Qatar 2022 resulted in much longer second halves. Consequently, the absolute distances covered by players in total and across most speed zones in the second half (i.e., m or km), were either similar to or even greater than the distances covered in the first half. To allow more appropriate comparisons to be drawn between halves, the physical data was adjusted to account for this and was represented as the relative distance covered per minute of match play (i.e., m · min^-1^). Collapsing all positional roles together resulted in a reduction of around 7% in the second half compared to the first in terms of the relative total distance covered. However, this reduction was more pronounced for defensive midfielders (9.8%), and least prominent for wide defenders (6.0%). Similarly, the relative distance covered at higher intensities (≥20 and ≥25 km · h^-1^) decline by 8–10% in the second half. Attacking midfielders demonstrated the most pronounced second half reduction at higher intensities (17.5–20.0%). Second half distance deficits have been reported in various domestic football competitions, albeit at much lower magnitudes than that reported in the present study [14, 23]. Although previous studies used broad positional role categories making direct comparisons challenging. Furthermore, these competitions did not adopt this new FIFA approach to added time that resulted in much longer games and particularly second halves. Thus, this additional time could have resulted in some fatigue across various positional roles. A multitude of mechanisms have been proposed to explain fatigue development in football, but researchers have failed to identify its precise cause [33]. Some have attributed this second-half decline to fatigue, as studies have reported depleted muscle glycogen stores at the end of a match and reduced creatine phosphate availability after intense periods [34]. However, football is a complex sport and second-half performance declines are impacted by much more than just fatigue because the changing game state and match importance can decrease or even increase a players physical output as well as many other factors [35].

Although this study was a detailed contemporary match analysis of the highest level of international football, it has numerous limitations that the reader should be cognisant of. Firstly, the data provider and not the author assigned the specialised positional roles which are based on the tactical systems adopted at the start of the game. Secondly, the physical data is limited to locomotor metrics and thus omits crucial information on position-specific acceleration and change of direction profiles [9, 36]. Moreover, one challenge for practitioners is linking each positional roles physical profile with various tactical phases or scenarios [11]. Unfortunately, this physical-tactical integration was not possible for this analysis thus limiting insights. Thus, at every opportunity the author layered the data with a contextual narrative to aid interpretation. Finally, this tournament offered a unique competition context compared to other tournaments that may have some impact on physical outputs and thus warrants further investigation (e.g., no long or medium-distance travel due to proximity of stadia, allocation of substitutes, more consolidated use of the Video Assistant Referee etc).

## CONCLUSIONS

The present data demonstrate the physical demands placed on various positional roles in contemporary international tournaments such as the FIFA World Cup Qatar 2022. Given the large variations in demands between and within each positional role, practitioners should ideally design high-intensity drills that replicate both position- and individual-specific characteristics that fully prepare players for the match demands. The reader should also be cognisant of how these positional trends align with team data from the tournament [37], so a more rounded view of match demands can be formulated.
